# Clinical Implications of Age in Differentiated Thyroid Cancer: Comparison of Clinical Outcomes between Children and Young Adults

**DOI:** 10.1155/2022/7804612

**Published:** 2022-02-21

**Authors:** Kwangsoon Kim, Sang-Wook Kang, Jandee Lee, Jong Ju Jeong, Kee-Hyun Nam, Woong Youn Chung

**Affiliations:** ^1^Department of Surgery, College of Medicine, The Catholic University of Korea, Seoul, Republic of Korea; ^2^Department of Surgery, Yonsei University College of Medicine, Seoul, Republic of Korea

## Abstract

**Background:**

Pediatric patients with differentiated thyroid cancer (DTC) present with unique characteristics compared to adult patients. This study aimed to evaluate clinical presentation and surgical outcomes according to age and to identify the clinical significance of age in DTC.

**Methods:**

In total, 98 pediatric patients, 1261 young adult patients, and 4017 adult patients with DTC who underwent thyroid surgery between January 1982 and December 2012 at Yonsei University Hospital (Seoul, Republic of Korea) were retrospectively reviewed. The mean follow-up duration was 120.4 ± 54.2 months.

**Results:**

Mean tumor size was significantly larger in the pediatric group than in the adult groups (*p* < 0.001). The recurrence rate was significantly higher in the pediatric group (14.3% versus 6.6% versus 3.0%, *p*=0.004 and *p* < 0.001). In multivariate analysis, the risk of disease-free survival (DFS) was lower in the adult group (HR, 0.362; *p* < 0.001). Reanalysis of patients with tumor size of 2–4 cm revealed that the adult group was not a significant risk factor for DFS in multivariate analysis (HR, 0.305; 95% CI, 0.158 to 0.588; *p* < 0.001).

**Conclusions:**

Our findings suggest that pediatric patients present with more aggressive features and higher recurrence rates compared to adult patients and should be carefully treated from initial evaluation to surgery and postoperative care.

## 1. Introduction

Thyroid cancer is the most common endocrine malignancy, and its global incidence has increased over the past two decades [1–4]. The general use of high-resolution ultrasonography has increased the identification of small nodules, which may be undetected by physical examination. Thyroid cancer during childhood is rare and accounts for 1.5% to 3.0% of all childhood cancers, but reports indicate that this incidence is increasing [2,5,6]. Papillary thyroid cancer (PTC) is the most common thyroid cancer in both pediatric and adult patients. Follicular thyroid cancer (FTC) is the second most common thyroid cancer but is rare during childhood [7,8].

Pediatric patients with differentiated thyroid cancer (DTC) present with several unique characteristics compared to adult patients. Compared to adult DTC, pediatric DTC tends to manifest as a more advanced disease at the time of diagnosis and is characterized by a more common extrathyroidal extension (ETE), lymph node metastasis, distant metastasis, and a higher risk of recurrence. Nevertheless, long-term prognosis has been reported to be better in pediatric patients than in adult patients [9–11].

Treatment of both pediatric and adult DTC comprises surgery, radioactive iodine (RAI) therapy, and thyroid-stimulating hormone (TSH) suppression therapy. According to the American Thyroid Association (ATA) management guidelines for children with DTC, the surgery of choice in pediatric DTC is total thyroidectomy (TT), which is preferred due to the more aggressive features of pediatric DTC, such as ETE and bilateral or multifocal disease [12].

The incidence of pediatric DTC increases with age and is more predominant in females. Most of the patients are diagnosed in adolescence, and adolescents between 15 and 19 years of age have a 10-fold higher incidence compared to the younger population [3, 13, 14]. Nevertheless, the differences in clinical presentation and clinical outcomes between pediatric and adult patients with DTC remain unclear. This retrospective study therefore aimed to evaluate the clinical presentation and surgical outcomes of patients with DTC according to age and to identify the clinical significance of age in patients with DTC. Our findings indicated that pediatric patients with DTC tended to exhibit a more aggressive clinical presentation. Further, we identified age as an independent risk factor for disease-free survival (DFS), underscoring the need to ensure careful and appropriate treatment of pediatric patients with DTC.

## 2. Materials and Methods

### 2.1. Patients

In total, 110 pediatric (<20 years old) patients, 1338 young adult patients (20–29 years old), and 4243 adult patients (30–39 years old) with DTC who underwent thyroid surgery at Yonsei University Hospital (Seoul, Republic of Korea) from January 1982 to December 2012 were retrospectively reviewed. In total, 12 pediatric patients, 77 young adults, and 226 adults were excluded owing to loss at follow-up and/or inadequate follow-up data. Baseline clinicopathological characteristics of study patients are presented in Table S1. All patients were analyzed by a complete review of medical charts and pathology reports. Of patients, 2620 (48.7%) underwent lobectomy and/or contralateral partial thyroidectomy (less than TT) with prophylactic or therapeutic ipsilateral central compartment neck dissection (CCND) and 2756 (51.3%) underwent TT with prophylactic or therapeutic ipsilateral CCND. Of patients who underwent TT, 691 (12.9%) underwent TT with therapeutic modified radical neck dissection (mRND) due to clinically suspicious or pathologically confirmed N1b nodes. The mean follow-up duration was 120.4 ± 54.2 months (range, 71–391). The study was conducted in accordance with the Declaration of Helsinki (as revised in 2013) and was approved by Yonsei University's institutional review board (IRB No.: 4-2019-0146), which waived the requirement for informed consent due to the retrospective nature of this study.

### 2.2. Postoperative Management and Follow-Up

All pediatric and adult patients were postoperatively managed according to the ATA management guidelines for children [12] and adults [15], respectively. RAI ablation was performed 4 to 6 weeks postoperatively using doses based on ATA guidelines. Whole-body scans (WBS) were performed after 5 to 7 days of RAI ablation in patients who underwent TT. Thyroglobulin (Tg) and antithyroglobulin antibody (TgAb) concentrations were assessed after TSH stimulation by T4 withdrawal or recombinant human TSH injection before RAI ablation. All patients received L-thyroxine with suppressive doses and were regularly followed up with physical examination, thyroid function tests, assessment of Tg and TgAb concentrations, and neck ultrasonography every 3 to 6 months and annually thereafter. Patients who presented with evidence of recurrence or distant metastasis on routine follow-up evaluations were assessed by additional diagnostic imaging, including computed tomography, positron emission tomography-computed tomography, and/or RAI WBS, to determine the location and extent of suspected recurrence. Disease recurrence was confirmed by imaging modalities and/or pathologic diagnosis using ultrasonography-guided fine-needle aspiration.

### 2.3. Statistical Analysis

Continuous and quantitative variables are presented as means and standard deviation (SD). Categorical and qualitative variables are reported as numbers with percentages. Student's *t*-test, chi-square test, or Wilcoxon rank-sum test was used for group comparisons. Univariate and multivariate Cox regression analyses were performed to identify independent predictors of DFS. Hazard ratios (HRs) with 95% confidence intervals (CIs) were calculated. DFS was analyzed for the three study groups using Kaplan-Meier survival analysis with a log-rank test. *P* < 0.05 was considered statistically significant. All statistical analyses were performed with the software package SPSS (version 23.0 for Windows; SPSS, Chicago, IL).

## 3. Results

### 3.1. Comparison among Different Age Groups

A total of 98 pediatric, 1261 young adult, and 4017 adult patients were enrolled in this study.  Table 1 presents the results of the comparison of baseline clinicopathological characteristics among the different age groups. The mean tumor size was significantly larger in the pediatric group than in the adult groups (2.1 ± 1.3 versus 1.3 ± 1.0 versus 1.0 ± 0.8, *p* < 0.001 and *p* < 0.001). The proportion of FTC was significantly higher in pediatric patients (7.1% versus 1.6% versus 0.7%, *p*=0.002 and *p* < 0.001). With regard to pathological T and N stages, the pediatric group exhibited a significantly higher grade. The recurrence rate was significantly higher in the pediatric group (14.3% versus 6.6% versus 3.0%, *p*=0.004 and *p* < 0.001). No significant differences were observed in gender, multifocality, bilaterality, or ETE among groups.  Table 2 presents the results of the comparison of management among the different age groups. The proportion of TT and mRND was significantly higher in the pediatric group than in the young adult and the adult groups (TT, *p* < 0.001 and *p* < 0.001; mRND; *p* < 0.001 and *p* < 0.001, respectively).

### 3.2. Risk Analysis for DFS in Different Age Groups

**Figure 1:**
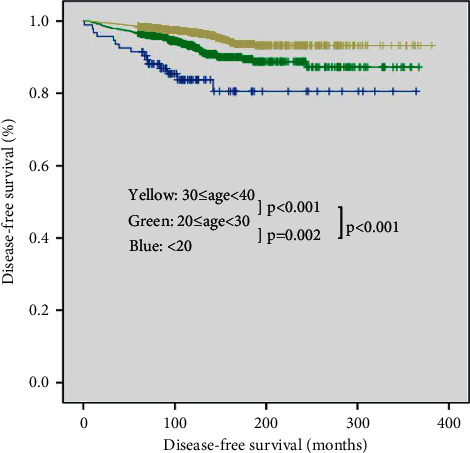
Disease-free survival curves of three groups (log rank; <20 versus 20–29 years, *p*=0.002; <20 versus 30–39 years, *p* < 0.001; 20–29 years versus 30–39 years, *p* < 0.001).

Univariate and multivariate Cox regression analyses were performed to identify independent risk factors for DFS (Table 3). In univariate analysis, the pediatric group was identified as a significant risk factor for DFS (young adult group: HR, 0.437; 95% CI, 0.252–0.757; *p*=0.003; adult group: HR, 0.210; 95% CI, 0.123–0.359; *p* < 0.001). Multivariate analysis revealed that the adult group had a significantly lower risk of DFS (HR, 0.362; 95% CI, 0.210–0.625; *p* < 0.001). Tumor size over 1 cm (HR, 2.272; 95% CI, 1.673–3.086; *p* < 0.001), N1a stage (HR, 2.074; 95% CI, 1.477–2.911; *p* < 0.001), and N1b stage (HR, 3.267; 95% CI, 2.256–4.732; *p* < 0.001) were identified as significant risk factors for DFS in multivariate analysis. Kaplan-Meier analysis revealed a significant difference in DFS between the pediatric group and adult groups (pediatric versus young adult group, *p*=0.002; pediatric versus adult group, *p* < 0.001; Figure 1).

### 3.3. Subanalysis of Pediatric Subgroups

Tables 4 and 5 present the results of subanalyses of clinicopathological characteristics and management in pediatric subgroups (≤16 years old or 17–19 years old). No significant differences were observed in tumor size, type of carcinoma, multifocality, bilaterality, or M stage between the two pediatric subgroups. The proportion of female patients was significantly higher in the older pediatric group than in the younger pediatric group (81.1% versus 93.4%, *p* < 0.001). The ETE was significantly greater in the younger pediatric group than in the older pediatric group (51.4% versus 27.9%, *p*=0.007). The younger pediatric group exhibited a significantly higher grade of pathological T and N stage compared to the older pediatric group (*p*=0.012 and *p* < 0.001). The proportion of TT and mRND was significantly higher in the younger pediatric group than in the older pediatric group (81.1% versus 52.5%, *p*=0.010, and 54.1% versus 16.4%, *p* < 0.001, respectively). In total, six (16.2%) patients in the younger pediatric group and eight (13.1%) patients in the older pediatric group were diagnosed with recurrence, but this difference was not significant (*p*=0.564).

### 3.4. Subanalysis of Older Pediatric Subgroup and Adult Groups

Tables 6 and 7 present the results of the subanalyses of clinicopathological characteristics and management of the three different groups over 16 years of age. No significant differences in most of clinicopathological characteristics were identified relative to the results presented in Table 1. However, the recurrence rate did not differ between the older pediatric and the young adult groups (13.1% versus 6.6%, *p*=0.064). The extent of operation and RAI therapy were not significantly different.

**Figure 2:**
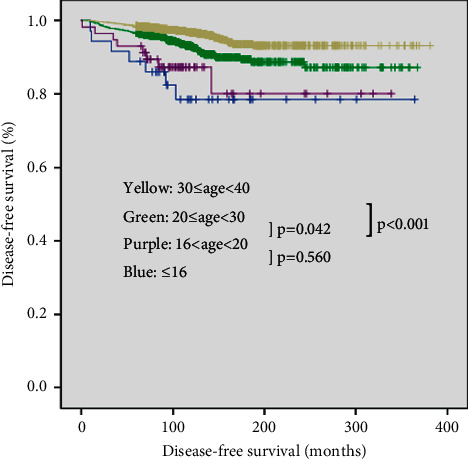
Disease-free survival curves of four groups (log rank; ≤16 years versus 17–19 years, *p*=0.560; 17–19 years versus 20–29 years, *p*=0.042; 17–19 years versus 30–39 years, *p* < 0.001).

Univariate and multivariate logistic regression analyses were performed to determine independent risk factors for DFS (Table 8). Results were similar to those presented in Table 3. Only the adult group was not identified as a significant independent risk factor for DFS in multivariate analysis (HR, 0.362; 95% CI, 0.206 to 0.627; *p*=0.006). In Kaplan-Meier analysis, no significant difference was observed in DFS between the younger pediatric and older pediatric groups (*p*=0.560; Figure 2). Significant differences in DFS were observed between the older pediatric group and adult groups (older pediatric versus young adult group, *p*=0.042; older pediatric versus adult group, *p* < 0.001; Figure 2).

### 3.5. Subanalysis of Patients in Different Age Groups with Tumor Size of 2 to 4 cm

Given that the majority of pediatric patients were diagnosed with palpable masses rather than by a screening test, we reanalyzed the patients with tumor sizes of 2 to 4 cm. In total, 47 pediatric, 187 young adult, and 367 adult patients were included in the reanalysis.  Table 9 presents the results of the comparison of clinicopathological characteristics among the three different age groups with tumor sizes of 2 to 4 cm. No significant differences were observed in the proportion of males, type of carcinoma, multifocality, bilaterality, ETE, and pathological T or M stage. The pediatric group presented with a significantly higher grade in the pathological N stage (*p*=0.006). Recurrence rate was significantly higher in the pediatric group (25.5% versus 16.6% versus 9.5%, *p*=0.001).

### 3.6. Risk Subanalysis for DFS in Patients in Different Age Groups with Tumor Size of 2 to 4 cm

**Figure 3:**
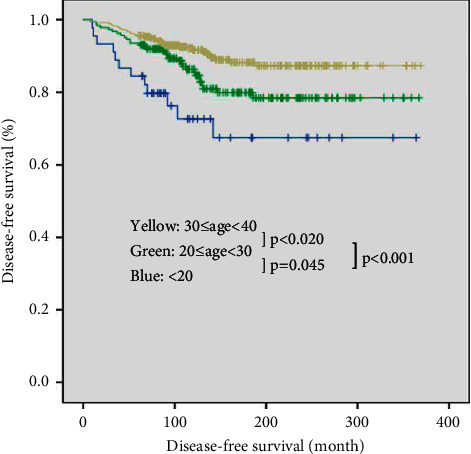
Disease-free survival curves of three groups with tumor size of 2 to 4 cm (log rank; <20 years versus 20–29 years, *p*=0.045; <20 years versus 30–39 years, *p* < 0.001; 20–29 years versus 30–39 years, *p*=0.020).

Univariate and multivariate logistic regression analyses were performed to determine independent risk factors for DFS in patients with tumor sizes of 2 to 4 cm (Table 10). Among the three different age groups, only the adult group was not a significant risk factor for DFS in both univariate and multivariate analyses (HR, 0.292; 95% CI, 0.151–0.563; *p* < 0.001; and HR, 0.305; 95% CI, 0.158–0.588; *p* < 0.001, respectively). Bilaterality (HR, 1.758; 95% CI, 1.061–2.913; *p*=0.029) was confirmed as a significant risk factor for DFS in multivariate analysis. Kaplan-Meier analysis revealed significant differences in DFS between the pediatric group and adult groups (pediatric versus young adult group, *p*=0.045; and pediatric versus adult group, *p* < 0.001; Figure 3).

## 4. Discussion

Pediatric and adult DTCs are considered distinct diseases, as the former is underscored by more aggressive features but a more favorable long-term prognosis [9,11,16]. Age is a major prognostic factor in DTC [17]. Therefore, the TNM staging system of DTC is classified according to an age of 55 years. Nevertheless, few studies have compared clinical presentation and surgical outcomes between pediatric and adult patients with DTC [18,19]. Here, we investigated the clinicopathological characteristics of 5376 patients with DTC to investigate the effects of age on surgical outcomes.

In this study, tumor size was larger in pediatric patients. Kim et al. reported that tumor size was larger in pediatric patients than in young adult patients [19]. However, no significant differences were observed in multifocal and bilateral disease between pediatric and adult groups, in contrast to previous reports [20–23]. Several reports have indicated that the incidence of multifocality in pediatric patients may reach 88% [24,25]. The ETE appeared to be more common in pediatric DTC, but we did not identify a significant difference in this regard. The thyroid gland is smaller in children than in adults, which may facilitate the occurrence of early capsular extension [26]. T, N, and M staging grade and the recurrence rate were significantly higher in the pediatric group than in the adult groups. These results were consistent with previous reports on the characteristics of pediatric DTC [27–30]. Nevertheless, the factors underpinning the more aggressive features of pediatric DTC have not been fully elucidated.

Based on the mean age of pediatric patients (16.7 years), we performed a subanalysis of the pediatric group. The ETE was more prevalent in the younger pediatric group (≤16 years old) than in the older pediatric group (17–19 years old). Further, the younger pediatric group presented with a significantly higher grade of T and N stage compared with the older pediatric group. These findings are consistent with previous reports [27,31,32]. Nevertheless, no significant differences were observed in multifocality, bilaterality, and ETE between adolescent and adult groups. Adolescent DTC tended to be similar to that in young adults, even the recurrence rate.

In multivariate analysis, only the adult group was not identified as a significant independent risk factor for DFS, suggesting that pediatric and young adult DTC have similar clinical outcomes. In addition, the frequency of intensive treatment, such as TT, mRND, and high-dose RAI therapy (>100 mCi), was significantly higher in the pediatric group than in the adult groups. These factors may have contributed to the similar prognosis of pediatric and young adult DTC.

In general, most of adult DTCs were diagnosed with a screening test, whereas most of pediatric DTCs were incidentally diagnosed. Physical examination is important given that palpable masses are more common in pediatric patients than in adult patients [10,33–35]. Indeed, 31–97% of pediatric patients with DTC present with a bulge in the anterior neck [4,10,36]. However, this manifestation is asymptomatic in childhood and is often noticed by parents or medical staff, rendering an early diagnosis of pediatric patients challenging. In this study, the mean tumor size of pediatric DTC was significantly larger than that of adult DTC. Further, we performed a subanalysis of clinicopathological characteristics in three different age groups with a tumor size of 2 to 4 cm. Despite adjusting for size differences, the recurrence rate was significantly higher in the pediatric group than in the adult groups (25.5% versus 16.6% versus 9.5%, *p*=0.001). Multivariate analysis revealed similar results to the preceding analyses. Our findings suggest that the prognosis of pediatric DTC is affected by other risk factors, such as age and bilaterality, in addition to tumor size. Jarzab et al. reported that younger children had a poorer prognosis compared to older children [32]. Additionally, Palmer et al. demonstrated that one of the best predictors of recurrence was multiple thyroid nodules at presentation [37].

The causative factors of differences in clinicopathological features and long-term outcomes between pediatric and adult DTC are obscure. One possible factor is differences in molecular pathogenesis between pediatric and adult DTC. Genetic studies of DTC indicate that the most common genetic defect is RET/PTC rearrangements in pediatric DTC, whereas BRAF^V600E^ and RAS mutations are most commonly detected in adult DTC [9, 38–40]. Additionally, the level of sodium-iodide symporter (*NIS*) gene expression is higher in pediatric DTC than in adult DTC [41, 42]. This implies greater responsiveness to RAI and better therapeutic outcomes in pediatric DTC than in adult DTC [42, 43].

According to the ATA management guidelines for children, TT is recommended in pediatric DTC due to the more aggressive features and increased risk of recurrence in pediatric DTC [12]. In addition, TT has several advantages, such as guaranteed RAI ablation and the use of Tg as a tumor marker. Nevertheless, TT is associated with various complications, including transient/permanent postoperative hypoparathyroidism, recurrent laryngeal nerve injury, and side effects due to lifetime TSH suppression. In this study, none of the pediatric patients developed permanent complications. Conservative surgical management is recommended to avoid surgical complications in pediatric patients with the nonadvanced disease. The optimal extent of operation for pediatric DTC remains under debate. The major controversial factors are the impact of extensive operations on recurrence and potential risks. Those who favor TT claim that this procedure is associated with improved DFS without significant complications if performed by experienced professionals. Handkiewicz et al. concluded that TT was able to remove all malignant tissue, decrease recurrence risk, and improve patient outcomes [31]. In contrast, those who favor conservative treatment claim that the less aggressive approach is associated with comparable surgical outcomes in selected patients and is considerably safer than TT. Gulcelik et al. advocated less aggressive treatment to decrease the risk of surgical complications [44]. Due to the low incidence, slow progression, and need for long-term follow-up to identify precise diagnosis in pediatric patients, the gold standard operative approach for pediatric DTC remains controversial.

A strength of this study is that all patients were treated and followed up with the same protocol comprising surgery, TSH suppression, RAI therapy, and use of imaging modalities by a multidisciplinary team. However, several limitations of the study should be addressed. First, the study design was retrospective in nature. Second, there may have been selection bias given that patient data were collected from a single tertiary institution and may not reflect the entire patient population. Third, of the study population, only 98 patients were in the pediatric group, which was significantly smaller than the other groups. Fourth, the mean follow-up period was relatively short (120.4 ± 54.2 months). This follow-up time limited our ability to compare long-term surgical outcomes between the three different age groups. Finally, only six (6.4%) pediatric patients were below 10 years of age, and the mean age of pediatric patients was 16.7 years. As such, this sample may not have been reflective of the entire pediatric population.

## 5. Conclusions

To the best of our knowledge, there is a paucity of studies comparing clinical presentation and surgical outcomes between pediatric and adult groups. This study demonstrated that pediatric patients presented with more aggressive features compared to adult patients. Further, the recurrence rate of DTC was higher in pediatric patients than in adult patients. Given that age was identified as an independent risk factor for DFS, our findings underscore the need for pediatric patients with DTC to be carefully treated from initial evaluation to surgery and postoperative care.

## Figures and Tables

**Table 1:** Comparison of clinicopathological characteristics among groups.

	<20 years old (A) (*n* = 98)	20–29 years old (B) (*n* = 1261)	30–39 years old (C) (*n* = 4017)	*p* value (A versus B)	*p* value (A versus C)
Age (years)	16.7 ± 3.0	26.2 ± 2.5	34.8 ± 2.8	<0.001	<0.001
Male : female	1 : 7.9	1 : 7.2	1 : 5.2	0.873	0.211
Male	11 (11.2%)	153 (12.1%)	650 (16.2%)		
Female	87 (88.8%)	1108 (87.9%)	3367 (83.8%)		
Tumor size (cm)	2.1 ± 1.3	1.3 ± 1.0	1.0 ± 0.8	<0.001	<0.001
Type of carcinoma				0.002	<0.001
PTC	91 (92.9%)	1241 (98.4%)	3989 (99.3%)		
FTC	7 (7.1%)	20 (1.6%)	28 (0.7%)		
Multifocality	24 (24.5%)	284 (22.5%)	975 (34.3%)	0.619	0.520
Bilaterality	15 (15.3%)	173 (13.7%)	592 (14.7%)	0.649	0.885
ETE	36 (36.7%)	359 (28.5%)	610 (15.2%)	0.402	0.153
T stage				<0.001	<0.001
T1	44 (44.9%)	815 (64.7%)	3248 (80.9%)		
T2	17 (17.3%)	85 (6.7%)	154 (3.8%)		
T3	33 (33.7%)	342 (27.1%)	558 (13.9%)		
T4	4 (4.1%)	19 (1.5%)	57 (1.4%)		
N stage				<0.001	<0.001
N0	25 (25.5%)	577 (45.8%)	2255 (56.2%)		
N1a	43 (43.9%)	454 (36.0%)	1331 (33.1%)		
N1b	30 (30.6%)	230 (18.2%)	431 (10.7%)		
M stage				0.064	0.018
M1	2 (2.0%)	4 (0.3%)	7 (0.2%)		
Recurrence	14 (14.3%)	83 (6.6%)	121 (3.0%)	0.004	<0.001

Data are expressed as patient's number (%) or mean ± SD. A statistically significant difference was defined as *p* < 0.05. PTC, papillary thyroid carcinoma; FTC, follicular thyroid carcinoma; ETE, extrathyroidal extension; T, tumor; N, node; M, metastasis.

**Table 2:** Comparison of management among groups.

	<20 years old (A) (*n* = 98)	20–29 years old (B) (*n* = 1261)	30–39 years old (C) (*n* = 4017)	*p* value (A versus B)	*p* value (A versus C)
Extent of operation				<0.001	<0.001
Less than TT	36 (36.7%)	605 (48.0%)	1979 (49.3%)		
TT	62 (63.3%)	656 (52.0%)	2038 (50.7%)		
Node dissection				<0.001	<0.001
CCND	68 (69.4%)	1031 (81.8%)	3586 (89.3%)		
mRND	30 (30.6%)	230 (18.2%)	431(10.7%)		
RAI therapy (mCi)				0.141	0.065
No	44 (44.9%)	670 (53.1%)	2189 (54.5%)		
30	30 (30.6%)	423 (33.5%)	1424 (35.5%)		
100–150	21 (21.4%)	147 (11.7%)	367 (9.1%)		
200	1 (1.0%)	19 (1.5%)	33 (0.8%)		
>200	2 (2.1%)	2 (0.2%)	4 (0.1%)		

Data are expressed as patient's number (%). A statistically significant difference was defined as *p* < 0.05. TT, total thyroidectomy; CCND, central compartment neck dissection; mRND, modified radical neck dissection; RAI, radioactive iodine.

**Table 3:** Univariate and multivariate analyses for DFS in different age groups.

	Univariate		Multivariate	
	HR (95% CI)	*p* value	HR (95% CI)	*p* value
Age (years)				
< 20	Ref.		Ref.	
20–29	0.437 (0.252–0.757)	0.003	0.599 (0.345–1.040)	0.069
30–39	0.210 (0.123–0.359)	<0.001	0.362 (0.210–0.625)	<0.001
Tumor size				
≤1 cm	Ref.		Ref.	
>1 cm	3.354 (2.509–4.484)	<0.001	2.272 (1.673–3.086)	<0.001
ETE	1.752 (1.332–2.303)	<0.001		
Multifocality	1.495 (1.124–1.988)	0.006		
Bilaterality	1.837 (1.346–2.508)	<0.001		
T stage				
T1	Ref.			
T2	2.267 (1.244–4.131)	0.008		
T3	1.950 (1.448–2.627)	<0.001		
T4	4.860 (2.564–9.213)	<0.001		
N stage				
N0	Ref.	-	Ref.	-
N1a	2.596 (1.862–3.619)	<0.001	2.074 (1.477–2.911)	<0.001
N1b	5.129 (3.613–7.283)	<0.001	3.267 (2.256–4.732)	<0.001
RAI therapy				
No	Ref.	-		
yes	1.709 (1.303–2.244)	<0.001		

Data are expressed as hazard ratio (HR) and 95% confidence interval (CI). A statistically significant difference was defined as *p* < 0.05. ETE, extrathyroidal extension; T, tumor; N, node; RAI, radioactive iodine.

**Table 4:** Subanalysis of clinicopathological characteristics in pediatric subgroups.

	≤16 years (*n* = 37)	17–19 years (*n* = 61)	*p* value
Age (years)	13.9 ± 3.1	18.4 ± 0.8	<0.001
Male : female	1 : 4.3	1 : 14.3	<0.001
Male	7 (18.9%)	4 (6.6%)	
Female	30 (81.1%)	57 (93.4%)	
Tumor size (cm)	2.3 ± 1.0	1.9 ± 1.5	0.205
Type of carcinoma			0.184
PTC	36 (97.3%)	55 (90.2%)	
FTC	1 (2.7%)	6 (9.8%)	
Multifocality	10 (27.0%)	14 (23.0%)	0.809
Bilaterality	8 (21.6%)	7 (11.5%)	0.247
ETE	19 (51.4%)	17 (27.9%)	0.007
T stage			0.012
T1	10 (27.0%)	34 (55.7%)	
T2	8 (21.6%)	9 (14.8%)	
T3	16 (43.2%)	17 (27.9%)	
T4	3 (8.1%)	1 (1.6%)	
N stage			<0.001
N0	7 (18.9%)	18 (29.5%)	
N1a	10 (27.0%)	33 (54.1%)	
N1b	20 (54.1%)	10 (16.4%)	
M stage			0.718
M1	1 (2.7%)	1 (1.6%)	
Recurrence	6 (16.2%)	8 (13.1%)	0.564

Data are expressed as patient's number (%) or mean ± SD. A statistically significant difference was defined as *p* < 0.05. PTC, papillary thyroid carcinoma; FTC, follicular thyroid carcinoma; ETE, extrathyroidal extension; T, tumor; N, node; M, metastasis.

**Table 5:** Subanalysis of management in pediatric subgroups.

	≤16 years (*n* = 37)	17–19 years (*n* = 61)	*p* value
Extent of operation			0.010
Less than TT	7 (18.9%)	29 (47.5%)	
TT	30 (81.1%)	32 (52.5%)	
Node dissection			<0.001
CCND	17 (45.9%)	51 (83.6%)	
mRND	20 (54.1%)	10 (16.4%)	
RAI therapy (mCi)			0.147
No	13 (35.1%)	31 (50.8%)	
30	12 (32.4%)	18 (29.5%)	
100–150	10 (27.0%)	11 (18.0%)	
200	0 (0%)	1 (1.6%)	
>200	2 (5.4%)	0 (0%)	

Data are expressed as patient's number (%). A statistically significant difference was defined as *p* < 0.05. TT, total thyroidectomy; CCND, central compartment neck dissection; mRND, modified radical neck dissection; RAI, radioactive iodine.

**Table 6:** Subanalysis of clinicopathological characteristics of groups over 16 years of age.

	17–19 years (A) (*n* = 61)	20–29 years old (B) (*n* = 1261)	30–39 years old (C) (*n* = 4017)	*p* value (A versus B)	*p* value (A versus C)
Age (years)	18.4 ± 0.8	26.2 ± 2.5	34.8 ± 2.8	<0.001	<0.001
Male : female	1 : 14.3	1 : 7.2	1 : 5.2	0.227	0.051
Male	4 (6.6%)	153 (12.1%)	650 (16.2%)		
Female	57 (93.4%)	1108 (87.9%)	3367 (83.8%)		
Tumor size (cm)	1.9 ± 1.5	1.3 ± 1.0	1.0 ± 0.8	<0.001	<0.001
Type of carcinoma				0.001	<0001
PTC	55 (90.2%)	1241 (98.4%)	3989 (99.3%)		
FTC	6 (9.8%)	20(1.6%)	28(0.7%)		
Multifocality	14 (23.0%)	284(22.5%)	975(34.3%)	0.938	0.881
Bilaterality	7 (11.5%)	173(13.7%)	592(14.7%)	0.848	0.586
ETE	17 (27.9%)	359 (28.5%)	610 (15.2%)	0.432	0.699
T stage				0.001	<0.001
T1	34 (55.7%)	815 (64.7%)	3248 (80.9%)		
T2	9 (14.8%)	85 (6.7%)	154 (3.8%)		
T3	17 (27.9%)	342 (27.1%)	558 (13.9%)		
T4	1 (1.6%)	19 (1.5%)	57 (1.4%)		
N stage				0.039	<0.001
N0	18 (29.5%)	577 (45.8%)	2255 (56.2%)		
N1a	33 (54.1%)	454 (36.0%)	1331 (33.1%)		
N1b	10 (16.4%)	230 (18.2%)	431 (10.7%)		
M stage				0.211	0.114
M1	1 (1.6%)	4 (0.3%)	7 (0.2%)		
Recurrence	8 (13.1%)	83 (6.6%)	121 (3.0%)	0.064	0.001

Data are expressed as patient's number (%) or mean ± SD. A statistically significant difference was defined as *p* < 0.05. PTC, papillary thyroid carcinoma; FTC, follicular thyroid carcinoma; ETE, extrathyroidal extension; T, tumor; N, node; M, metastasis.

**Table 7:** Subanalysis of treatment management of groups over 16 years of age.

	17–19 years (A) (*n* = 61)	20–29 years old (B) (*n* = 1261)	30–39 years old (C) (*n* = 4017)	*p* value (A versus B)	*p* value (A versus C)
Extent of operation				0.126	0.153
Less than TT	29 (47.5%)	605 (48.0%)	1979 (49.3%)		
TT	32 (52.5%)	656 (52.0%)	2038 (50.7%)		
Node dissection				0.967	0.092
CCND	51 (83.6%)	1031 (81.8%)	3586 (89.3%)		
mRND	10 (16.4%)	230 (18.2%)	431(10.7%)		
RAI therapy (mCi)				0.851	0.351
No	31 (50.8%)	670 (53.1%)	2189 (54.5%)		
30	18 (29.5%)	423 (33.5%)	1424 (35.5%)		
100–150	11 (18.0%)	147 (11.7%)	367 (9.1%)		
200	1 (1.6%)	19 (1.5%)	33 (0.8%)		
>200	0 (0%)	2 (0.2%)	4 (0.1%)		

Data are expressed as patient's number (%). A statistically significant difference was defined as *p* < 0.05. TT, total thyroidectomy; CCND, central compartment neck dissection; mRND, modified radical neck dissection; RAI, radioactive iodine.

**Table 8:** Univariate and multivariate analyses for DFS in different age groups.

	Univariate		Multivariate	
	HR (95% CI)	*p* value	HR (95% CI)	*p* value
Age (years)				
17–19	Ref.		Ref.	
20–29	0.477 (0.242–0.826)	0.046	0.561 (0.337–1.117)	0.156
30–39	0.230 (0.110–0.387)	<0.001	0.362 (0.206–0.627)	0.006
Tumor size				
≤1 cm	Ref.		Ref.	
>1 cm	3.228 (2.411–4.648)	<0.001	2.417 (1.594–3.163)	<0.001
ETE	1.704 (1.295–2.412)	<0.001		
Multifocality	1.427 (1.103–1.864)	0.006		
Bilaterality	1.801 (1.294–2.576)	<0.001	1.470 (0.954–1.932)	0.026
T stage				
T1	Ref.			
T2	2.346 (1.216–4.215)	0.005		
T3	1.936 (1.426–2.518)	<0.001		
T4	4.195 (2.286–8.639)	<0.001		
N stage				
N0	Ref.	-	Ref.	-
N1a	2.692 (1.749–3.943)	<0.001	2.349 (1.256–2.984)	<0.001
N1b	5.095 (3.437–7.842)	<0.001	4.046 (2.547–5.438)	<0.001
RAI therapy				
No	Ref.	-		
yes	1.785 (1.203–2.548)	<0.001		

Data are expressed as hazard ratio (HR) and 95% confidence interval (CI). A statistically significant difference was defined as *p* < 0.05. ETE, extrathyroidal extension; T, tumor; N, node; RAI, radioactive iodine.

**Table 9:** Subanalysis of clinicopathological characteristics of patients with tumor size of 2 to 4 cm.

	<20 years (*n* = 47)	20–29 years (*n* = 187)	30–39 years (*n* = 367)	*p* value
Age (years)	15.6 ± 3.7	25.9 ± 2.6	34.3 ± 2.8	<0.001
Male : female	1 : 4.9	1 : 5.9	1 : 4.2	0.374
Male	8 (17.0%)	27 (14.4%)	70 (19.1%)	
Female	39 (83.0%)	160 (85.6%)	297 (80.9%)	
Tumor size (cm)	2.8 ± 0.7	2.7 ± 0.6	2.6 ± 0.6	0.321
Type of carcinoma				0.859
PTC	45 (95.7%)	178 (95.2%)	352 (95.9%)	
FTC	2 (4.3%)	9 (4.8%)	15 (4.1%)	
Multifocality	15 (31.9%)	42 (22.5%)	98 (26.7%)	0.385
Bilaterality	13 (27.7%)	30 (16.0%)	73 (19.9%)	0.231
ETE	24 (51.1%)	92 (49.2%)	192 (52.3%)	0.572
T stage				0.357
T2	20 (42.6%)	93 (49.7%)	172 (46.9%)	
T3	23 (48.9%)	85 (45.5%)	175 (47.7%)	
T4	4 (8.5%)	9 (4.8%)	20 (5.4%)	
N stage				0.006
N0	10 (21.3%)	67 (35.8%)	114 (31.1%)	
N1a	14 (29.8%)	62 (33.2%)	154 (42.0%)	
N1b	23 (48.9%)	58 (31.0%)	99 (26.9%)	
M stage				0.260
M1	2 (4.3%)	0 (0%)	4 (1.1%)	
Recurrence	12 (25.5%)	31 (16.6%)	35 (9.5%)	0.001

Data are expressed as patient's number (%) or mean ± SD. A statistically significant difference was defined as *p* < 0.05. PTC, papillary thyroid carcinoma; FTC, follicular thyroid carcinoma; ETE, extrathyroidal extension; T, tumor; N, node; M, metastasis.

**Table 10:** Univariate and multivariate analyses of DFS in three different age groups with tumor size of 2 to 4 cm.

	Univariate		Multivariate	
	HR (95% CI)	*p* value	HR (95% CI)	*p* value
**Age (years)**				
< 20	Ref.		Ref.	
20–29	0.514 (0.264–1.002)	0.051	0.554 (0.283–1.085)	0.085
30–39	0.292 (0.151–0.563)	<0.001	0.305 (0.158–0.588)	<0.001
**Bilaterality**	1.770 (1.072–2.922)	0.026	1.758 (1.061–2.913)	0.029
**N stage**				
N0	Ref.	-		
N1a	1.468 (0.818–2.634)	0.198		
N1b	2.117 (1.178–3.805)	0.012		

Data are expressed as hazard ratio (HR) and 95% confidence interval (CI). A statistically significant difference was defined as *p* < 0.05. N, node.

## Data Availability

The data that support the findings of this study are available upon request from the corresponding author. The data are not publicly available due to privacy or ethical restrictions.

## Supplementary Materials

Supplementary Materials Table S1: baseline clinicopathological characteristics of the study patients.
