# Supplementary material for: Clinical Implications of Age in Differentiated Thyroid Cancer: Comparison of Clinical Outcomes between Children and Young Adults
Source: Int J Endocrinol. 2022 Feb 21;2022:7804612. doi: 10.1155/2022/7804612 (PMC8885294; doi:10.1155/2022/7804612)
Supplement: Supplementary Materials — Table S1: baseline clinicopathological characteristics of the study patients. [file 7804612.f1.docx]

**Table S1.** Baseline clinicopathologic characteristics of the study patients

| **Total 5376 patients** | |
| --- | --- |
| **Age (years)** | 32.5 ± 5.0  (range, 5-39) |
| < 20 | 98 (1.8%) |
| 20 ≤ age < 30 | 1261 (23.5%) |
| 30 ≤ age < 40 | 4017 (74.7%) |
| **Male : Female** | 1: 5.6 |
| Male | 814 (15.1%) |
| Female | 4562 (84.9%) |
| **Tumor size (cm)** | 1.1 ± 0.9  (range, 0.2-10.0) |
| **Type of carcinoma** |  |
| PTC | 5321 (99.0%) |
| FTC | 55 (1.0%) |
| **Multifocality** | 1283 (23.9%) |
| **Bilaterality** | 780 (14.5%) |
| **ETE** | 1005 (18.7%) |
| **T stage** |  |
| T1 / T2 / T3 / T4 | 4107 (76.4%) / 256 (4.8%) / 933 (17.3%) / 80 (1.5%) |
| **N stage** |  |
| N0 / N1a / N1b | 2857 (53.1%) / 1828 (34.0%) / 691 (12.9%) |
| **M stage** |  |
| M1 | 13 (0.2%) |
| **Extent of operation** |  |
| Less than TT | 2620 (48.7%) |
| TT | 2756 (51.3%) |
| **Node dissection** |  |
| CCND | 4685 (87.1%) |
| mRND | 691 (12.9%) |
| **RAI therapy (mCi)** |  |
| No | 2903 (54.0%) |
| 30 /100-150 / 200/ >200 | 1877 (34.9%) / 535 (9.9%) / 53 (1.1%) / 8 (0.1%) |
| **Recurrence** | 218 (4.1%) |
| **Follow up duration (months)** | 120.4 ± 54.2  (range, 71-391) |

Data are expressed as patient’s number (%), or mean ± SD.

Abbreviations: PTC, papillary thyroid carcinoma; FTC, follicular thyroid carcinoma; ETE, extrathyroidal extension; T, tumor; N, node; M, metastasis, TT, total thyroidectomy; CCND, central compartment neck dissection; mRND, modified radical neck dissection; RAI, radioactive iodine
